# Puffball spores improve wound healing in a diabetic rat model

**DOI:** 10.3389/fendo.2022.942549

**Published:** 2022-08-31

**Authors:** Tangtang He, Pengfei Sun, Bo Liu, Shiwei Wan, Penghua Fang, Jun Chen, Guicheng Huang, Wen Min

**Affiliations:** ^1^ Department of Bone Injury of Traditional Chinese Medicine, Affiliated Hospital of Nanjing University of Chinese Medicine, Nanjing, China; ^2^ Key Laboratory for Metabolic Diseases in Chinese Medicine, First College of Clinical Medicine, Nanjing University of Chinese Medicine, Nanjing, China; ^3^ First Clinical Medical College, Nanjing University of Chinese Medicine, Nanjing, China; ^4^ Jiangsu Provincial Engineering Research Center of Traditional Chinese Medicine External Medication Development and Application, Nanjing University of Chinese Medicine, Nanjing, China

**Keywords:** diabetic wound ulcer, Puffball spores, oxidative stress, wound healing, angiogenesis

## Abstract

Persistent chronic oxidative stress is a primary pathogenic characteristics of diabetic foot ulcers. Puffball spores are a traditional Chinese medicine used to treat diabetic foot ulcers infections and bedsores. However, their effects against diabetic wounds and the mechanism underlying these effects remain largely unknown. The present study explored the effectiveness of puffball spores in diabetic wound treatment and the mechanisms underlying their effects. Sprague-Dawley rats with streptozotocin (STZ)-induced diabetes were treated with puffball spores to ascertain whether they accelerated wound healing.Real-time quantitative PCR, western blotting, hematoxylin-eosin and Masson’s trichrome staining, immunohistochemistry analysis, and immunofluorescence assays were performed. As indicated by wound and serum histology and biochemical analyses, the puffball spores accelerated wound healing by activating Akt/Nrf2 signaling and promoting the expression of its downstream antioxidant genes, markedly stimulating antioxidant activity and enhanceing angiogenesis and collagen deposition. Our findings showed that puffball spores could accelerate diabetic wound healing, enhance antioxidant ability, promote the expression of vascular markers, and suppress inflammation, thus providing a theoretical basis for the treatment of diabetic and refractory wounds.

## Introduction

Diabetic foot ulcers (DFUs) are a common complication of diabetes. It is a significant health concern worldwide with a challenging clinical treatment (1, 2). This highly preventable diabetes complication is the primary cause of lower-extremity amputations (3). A study found that patients with DFUs had a much 2.5-fold higher risk of death than patients without lower extremity wounds, with an estimated 5-year mortality rate of 42% for patients with DFUs (4). Although various drugs are availabled for DFUs, the ulcers usually remain refractory and impact the quality of life of patients (5). Therefore, it is essential to investigate new strategies to accelerate healing of these cutaneous ulcers.

Wound healing is a complex process,that can be divided into main four progressive stages: hemostasis, inflammation, proliferation, and remodeling (6). Compared with non-diabetic wounds, the diabetic wound healing process has a prolonged the inflammatory stage, with the excessive secretion of inflammatory cytokines, such as tumor necrosis factor-alpha (TNF-α), interleukin(IL)-1β, and IL-6. This leads to increased tissue damage, which delays diabetic wound healing by interfering with the proliferation and maturation stages (7). In long-term hyperglycemia, accumulated advanced glycation end products (AGEs) increase reactive oxygen species (ROS) production (8). Excessive free radicals and ROS production in wound tissue can lead to an imbalance between oxidation and antioxidation. ROS, including anion superoxide, hydroxyl radicals, peroxide radicals, and nitric acid radicals, are key signaling molecules contributing to inflammatory disease progressions (9). As a result, wound healing results in a prolonged inflammatory response and a severe angiogenesis disorder. In patients with diabetic wounds, prolonged inflammation and blocked angiogenesis are the main reasons why taking a longer healing time than that for patients with normal wounds. Maintaining oxidative stress balance is favorable for accelerating diabetic wound healing.

Puffball is a traditional Chinese medicine, commomly used to trean several diseases such as hemostasis, throat pain, and cough (10). Puffball is mostly used as wound dressings, because dry, mature spores have a positive effect on wound hemostasis. Although it has been confirmed to possess effective clinical value, its active components and monomers have only recently been separated and identified. Some puffball extracts exert anti-cancer activity (11). Puffball extracts were shown to decrease the formation of nitric oxide (NO) in RAW264.7 macrophages activated with lipopolysaccharide (LPS), eliciting an anti-inflammatory effect (12). In addition, Puffball extracts have also shown scavenging potential, reducing the levels of free radicals and enhancing antioxidative action (13).

Growing evidence indicates that puffball spores have positive effects on diabetic wounds. Nuclear factor erythroid 2-related factor 2 (Nrf2) knockout mice with diabetses showed delayed wound healing, accompanied by oxidative damage (14). However, it remains unclear whether puffball spores promote diabetic wound healing by promoting antioxidant enzyme activity and activating the AKT/Nrf2 signaling pathway to enhance antioxidation. Further studies of oxidative stress markers and related signaling pathways are needed to clarify the mechanism underlying the action of these spores. We created a diabetic animal model *in vivo*. To examine the therapeutic effects of puffball spores on diabetes wound healing and to investigate the machanisms underlying their anti-inflammatory, angiogenesis, and antioxidation mechanisms.

## Materials and methods

### Preparation of puffball spores

Puffball was purchased from Anhui Wansheng Chinese Herbal Pieces Ltd (cat.No.190801, Fuyang, China). The puffball was accurately weighed, and the internal spores were separated into a clean mouth container. This was, then passed through no. 5 and 6 sieves. The puffball spores were autoclaved in a wide-mouth bottle at a pressure of 103.4kPa and an internal temperature of 121.3°C for 15–30 minutes.

### Animals

Sixty male Sprague-Dawley (SD) rats (weight, 320–400 g; age, 12 weeks) were purchased from Qing Long Mountain Company of Lab Animal Breeding (Certificate number: SCXK (Zhe) 2019-0002 Nanjing, China). These SD rats were divided into blank group and experimental group.

After the completion of modeling, the experimental group was randomly divided into the following groups: puffball spore, Ag+ antibacterial gel (positive group), and model groups. The Animal Ethics Committee of the Nanjing University of Chinese Medicine approved the animal experiments (No. 202009A034).

We collected 5 mL of blood from all the rats under anesthesia after they had fasted for 12 hours. The serum was separated by centrifuging the blood at 3500 RPM for 15 minutes. Wound samples were obtained using a skin-biopsy punch. The wound areas were sampled for hematoxylin-eosin (HE), Masson’s trichrome (MT), immunofluorescence (IF), Immunohistochemistry (IHC), Real-time quantitative (RT-qPCR), and western blot (WB) analyses.

### Establishment of a diabetic rat model

After 2 weeks of adaptive feeding, the rats from the model, puffball spore, and Ag+ groups were fed a high-fat diet for 6 weeks. Next, streptozotocin (STZ; 40 mg/kg, Sigma, USA, Cat. No. S0130) in citrate buffer (pH 4.5) was injected intraperitoneally to induce diabetes. Glucose levels were measured using a glucometer three days after diabetes induction (Sinocare, Changsha, China). Diabetes was defined as blood glucose levels > 16.7 mmol/l. Weekly blood glucose testing and body weight measurements were performed to ensure that diabetes status remained stable.

### Establishment of a diabetic wound model

All groups established a wound model. After anesthesia with sodium pentobarbital (45 mg/kg), the hair of the hind legs was shaved. Each animal received a single, round, full-thickness wound (10 mm) on a hind leg.After modeling was complete, puffball spores and Ag+ antibacterial gel were evenly applied to the wound at the dosage of 1 g/cm^2^, changed once a day. Rats in the model and blank groups received the same amount of physiological salt. The wounds were wrapped and fixed with sterile gauze for 14 consecutive days.

### Measurement of wound healing area

A series of wound changes were recorded and analyzed using ImageJ on days 0, 3, 7, and 14 (NIH, USA). The wound healing ability was quantified by the following formula: wound healing ability = (F_0_-F_3, 7, 14)_/F_0_ × 100%, where F_0_ represents the primary wound area, and F_3, 7, 14_ represents the wound area on days 3, 7, and 14 respectively.

### Histological examination

Sterile gauze was used to cover the entire wound area, until the samples were harvested on day 14. The harvested samples were fixed in 10% neutral formalin (Chunyu, China) and embedded in paraffin for histological analysis. The wound tissues were sectioned with a microtome (at a thickness of 5 mm, Leica, Germany) and dehydrated in a graded ethanol series for routine HE (Solarbio, Beijing, China, Cat. No. G1120) and MT (Solarbio, Beijing, China, Cat. No. G1340) staining to detect for collagen fibers. The HE and MT sections were observed under a light microscope (Nanjing Keygen, Nanjing, China).

### Immunohistochemistry assay

The IHC method was used to test the *in situ* CD31, pAKT, Nrf2 protein expression in the wound tissues of the rats. Briefly, the paraffin tissue sections were obtained as described above. Subsequently, the paraffin sections (5 mm thick) were deparaffinized, blocked with 5% blocking buffer for 50 minutes, incubated with anti-CD31 antibodies (1:200, Servicebio, Wuhan, China, Cat. No. GB11063-2), anti-pAKT antibodies(1:200,

CellSignalingTechnology, USA, Cat. No. 4060S), anti-Nrf2 antibodies(1:500, Wanleibio, Shenyang, China, Cat. No.WL02135) and observed under a light microscope (Nanjing Keygen, Nanjing, China).

### Enzyme-linked immunosorbent assay

The serum levels of inflammatory, oxidative stress, and angiogenic factors were detected using ELISA. Following the manufacturer’s instructions, ELISA kits were used to measure the levels of IL-1β (MLBio, Shanghai, China, Cat. No. ml037361), TNF-α (MLBio, Shanghai, China, Cat. No. ml002859), 8-hydroxy 2 deoxyguanosine(8-oxo-dG, MLBio, Shanghai, China, Cat. No. ml059056), and vascular endothelial growth factor (VEGF) (MLBio, Shanghai, China, Cat. No. ml064294). The final concentrations of these factors were determined using a microplate reader.

### Immunofluorescence assay

Following the manufacturer’s instructions, after being deparaffinized and blocked, the paraffin sections (5 mm thick) were incubated with anti-ROS primary antibodies (1:200, Servicebio, Wuhan, China, Cat. No. GDP1018) and then with fluorescein isothiocyanate (FITC)-conjugated secondary antibodies (Servicebio, Wuhan, China, Cat. No. GB22302). Finally, diamidino-phenyl-indole (DAPI) (Servicebio, Wuhan, China, Cat. No. G1012) was used to stain the cell nuclei. A fluorescence microscope (IX2-SL 81; Olympus) was used to capture the images.

### Detection of oxidative stress-related factors

Malondialdehyde(MDA) levels, superoxide dismutase (SOD) activity, catalase (CAT) activity, glutathione peroxidase (GSH-Px) activity, and total antioxidant capacity (T-AOC) were detected using the MDA (Solarbio, Shanghai, China, Cat. No.A003-1), SOD (NJJCBIO, Nanjing, China, Cat. No.A001-3), CAT (A007-1-1, NJJCBIO, Nanjing, China, Cat. No.A007-1), GSH-Px (NJJCBIO, Nanjing, China, Cat. No.A005), and T-AOC (Beyotime, Shanghai, China, Cat. No.A015-3-1) assay kits, respectively. The working solution and the sample were mixed and added to 96-well plates, the absorbance of the samples was measured. Calculations were done as per the instructions.

### Total RNA extraction and real-time PCR

Following the manufacturer’s instructions, RNA was isolated from wound tissues with TRIzol reagent (Vazyme, Nanjng, China, Cat. No. R401-01). The wound tissues were mechanically disaggregated using the TissueLyser apparatus (Retsch, MM 400, Shanghai, China) for 60 seconds at 30 Hz. After a 5-minute centrifugation, the supernatant was collected for further analysis. An Applied Biosystems 7500 instrument was used to detect mRNA using real-time quantitative PCR. The PCR data were analyzed using the 2−ΔΔCT method.

### Western blotting analysis

The total wound tissue proteins were extracted using radio-immunoprecipitation assay (RIPA) lysis buffer (Thermo Fisher Scientific, USA, Cat. No. 01408/30450) and quantified using the BCA Protein Assay Kit (Yeasen Biotechnology, Shanghai, China, Cat. No.P0010). The PVDF membranes were blocked with 5% blocking buffer for 1 hour, and incubated overnight at 4°C with antibodies against AKT(1:5000, Proteintech, Wuhan, China, 60203-2-Ig), pAKT(1:2000, Cell Signaling Technology, USA, Cat. No.31957), Nrf2(1:500, Wanleibio, Shenyang, China, Cat. No.WL02135), heme oxygenase-1 (HO-1, 1:500, Wanleibio, Shenyang, China, Cat. No.WL02400), NAD(P)H:quinone oxidoreductase-1 (NQO1, 1:500, Wanleibio, Shenyang, China, Cat. No.WL04860), and β-actin (1:4000, Proteintech, Wuhan, China, Cat. No. 20536-1-AP). Finally, the proteins bands were developed with Image Quant LAS 4000 mini and analyzed using Image J.

### Statistical analysis

The SPSS 25.0 software was used to analyze the data. The data from each group were analyzed using one-way ANOVA analysis of variance. Data were presented as the mean ± SEM; differences with P values< 0.05 were considered statistically significant.

## Results

### Puffball spore treatment enhances wound healing

**Figure 1:**
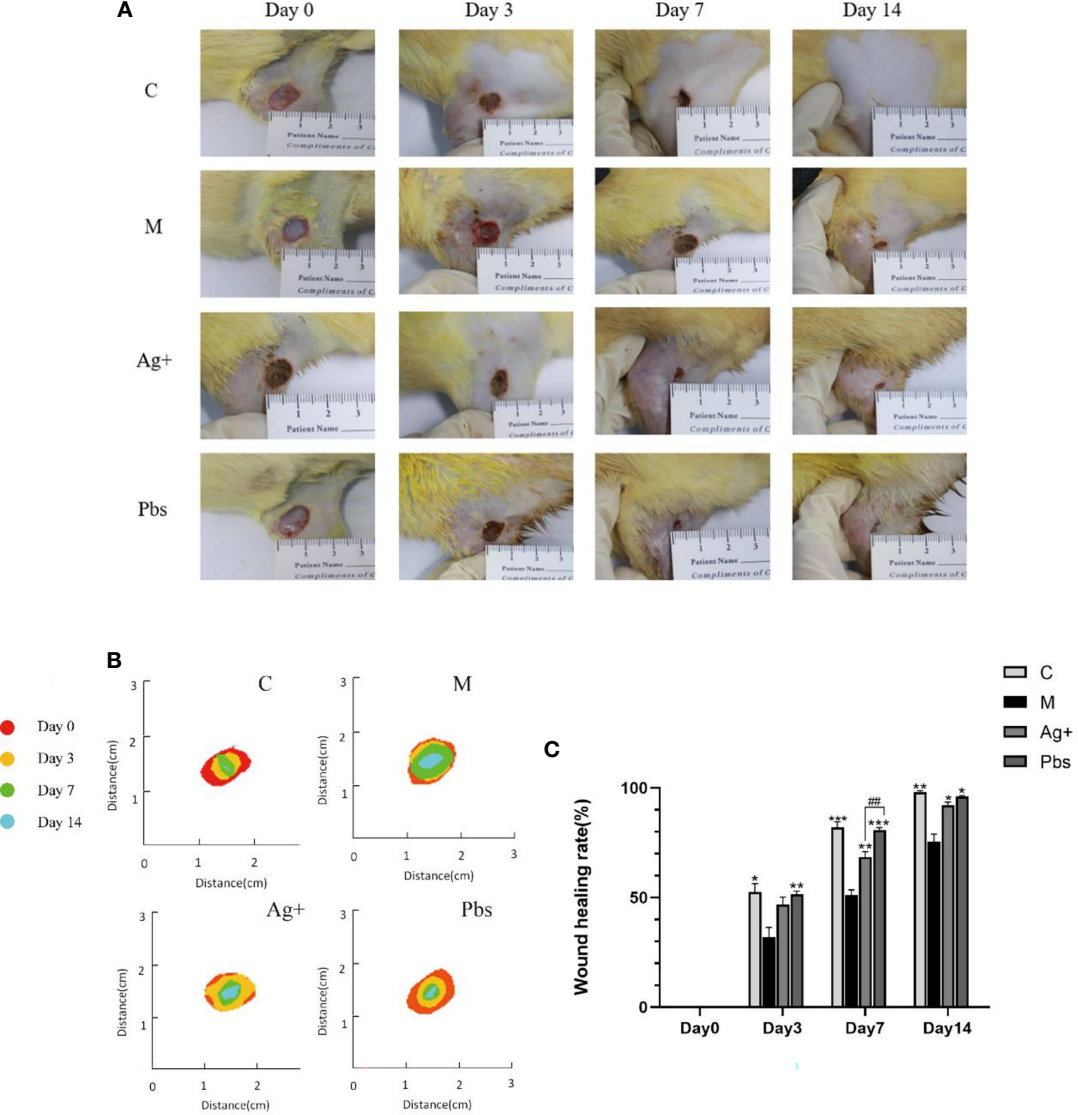
The effects of wound healing by puffball spore treatment in diabetic rats. **(A)** The wound areas were recorded by a camera on days 0, 3, 7, and 14. **(B)** Heat map of the wound healed by different treatments over 14 days. **(C)** Quantitative data of relative wound area at different time points.(Means ± SEM, n = 7; *P < 0.05, **P < 0.01, ***P < 0.001 vs. Model group, ##P < 0.01 vs. positive group.).

An *in vivo* wound-healing study of puffball spores was performed in STZ-induced diabetic rats using the excision wound method at different time intervals. To test the puffball spore wound-healing efficacy, wound areas were measured on days 0, 3, 7, and 14. The wound area gradually decreased over time. The puffball spores and Ag+ antibacterial gel exhibited a stronger wound-healing effect than that observed in the model groups (**Figures 1A–C**). The wound healed most rapidly on day 7.On day 3, the wound healing rates in the four groups were 52.48%, 31.39%, 46.49%, and 51.46%, respectively. On day 7, the wound healing rate of the model group was 51.20%, while those of the other three groups were 82.16%, 68.57%, and 80.82%, respectively. After 14 days, the wound healing rate in the model group was as high as 75.49%, and that in the puffball spore group was 96.13% (*P< 0.05).

### Histological observation of wound healing process

**Figure 2:**
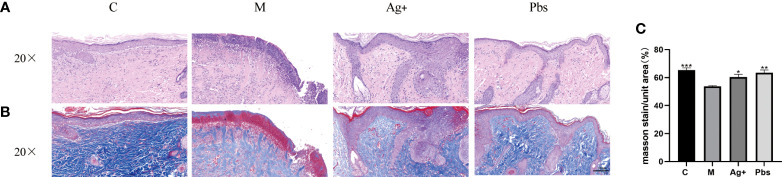
Puffball spores improved diabetic foot ulcer recovery in rats. **(A)** Representative HE staining images of wound tissues, (scale bar = 100 µm; magnification, 200×; n = 3). **(B)** Representative images of Masson’s trichrome (MT) staining, (scale bar = 100 µm; magnification, 200×; the blue color represents collagen; n = 3). **(C)** Wound tissue collagen fiber quantification using Image-Pro Plus. (Means ± SEM, n = 3, *P < 0.05, **P < 0.01, ***P<0.001 vs. model group).

The wound tissue harvested on day 14 were used for HE and Masson. Compared with the model group, the puffball spore group showed a greater reduction in cutaneous tissue damage, according to the histological analysis (**Figures 2A, B**). We also observed that wounds treated with puffball spore solution exhibited better granulation formation, collagen deposition, and denser alignment (**Figure 2C**).

### Puffball spores accelerate angiogenesis and reduce the expression of inflammatory factors

**Figure 3:**
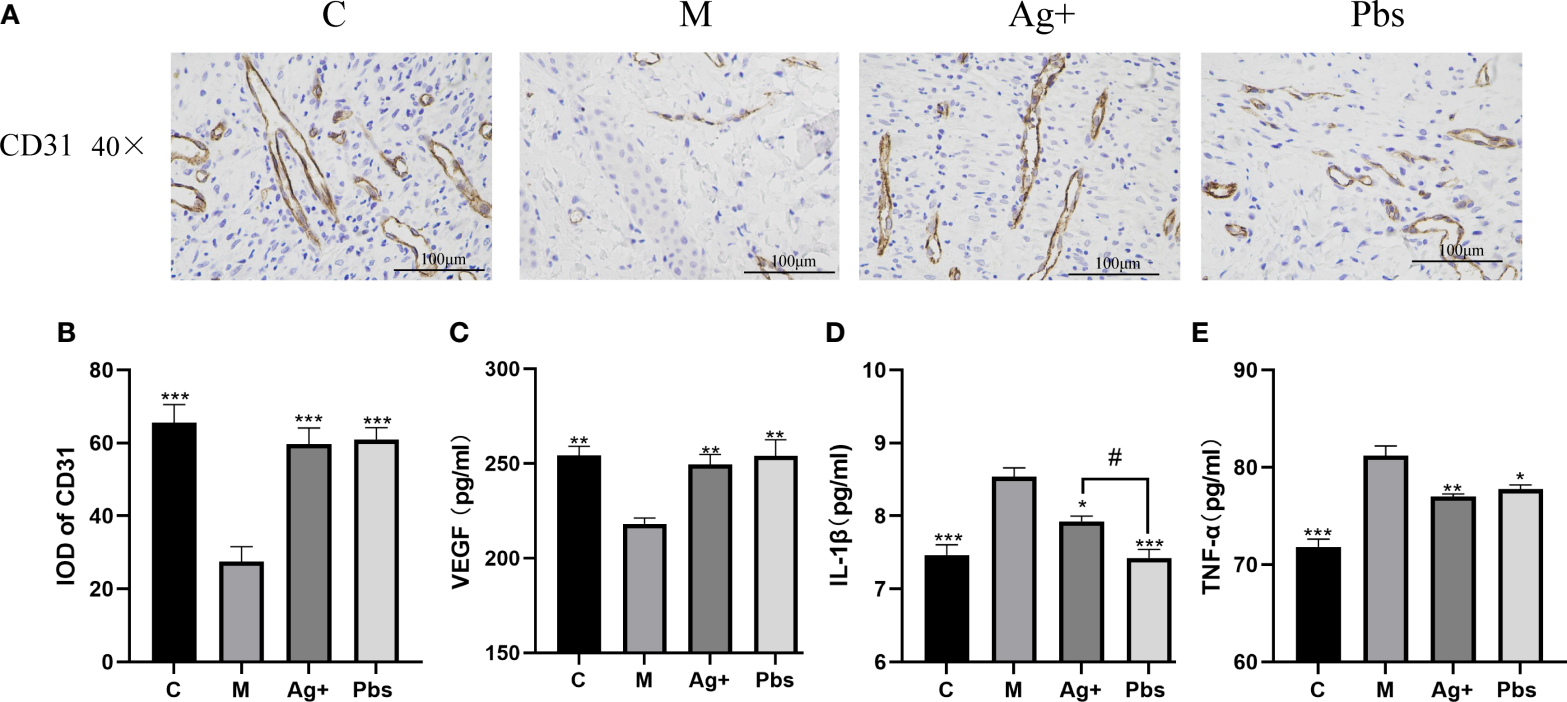
Effects of puffball spores on cytokine production in the serum of diabetics rats. **(A)** CD31 expression in wound tissues was detected by immunohistochemistry (scale bar = 100 µm; magnification, 400×; n = 3). **(B)** Quantitative data of wound tissue CD31 expression using IPP. (Means ± SEM, n = 3, **P < 0.01, ***P < 0.001 vs. model group). **(C)** VEGF, **(D)** IL-1β and **(E)**TNF-α were assayed by ELISA. (Means  ±  SEM, n  =  6, *P < 0.05, **P < 0.01 vs. model group; #P < 0.05 vs. positive group).

Angiogenesis is an essential wound healing process. CD31 indicates angiogenesis level. We employed immunohistochemical staining to measure CD31 protein expression in diabetic rats to determine whether puffball spores can stimulate angiogenesis. **Figures 3A, B** show that expression of CD31 in newly healed skin tissue within 14  days of treatment, compared to the case in model group. Using the ELISA method, we detected the expression of inflammatory factors IL-1β, TNF-α, and VEGF in diabetic rat serum. One of the main causes of poor neovascularization and delayed wound healing is the loss of VEGF.As shown in **Figure 3C**, puffball spore treatment increased VEGF expression in newly healed wound tissue within 14  days. In contrast, the IL-1β and TNF-α serum levels were lower than those in the model group (**Figures 3D, E**).

### Puffball spores increase ROS scavenging and protect against oxidative damage

**Figure 4:**
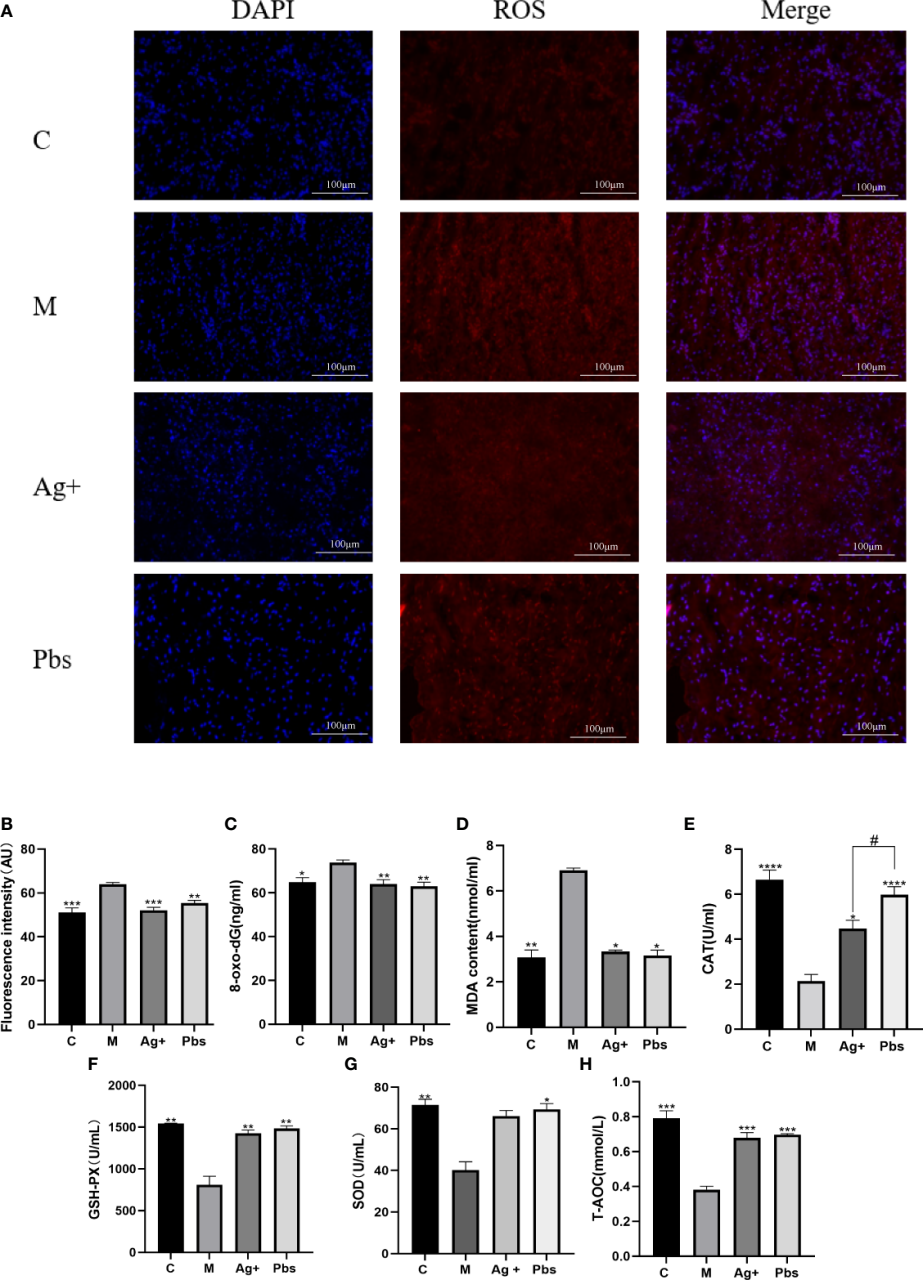
Puffball spores reduced oxidative damage by increasing antioxidant capacity. **(A, B)** Immunofluorescent staining reveals a decrease in the fluorescence intensity in the samples from the puffball spore-treatments group (scale bar = 100 µm; magnification, 400×; n = 3). **(C)** 8-oxo-dG, **(D)** MDA, **(E)** CAT, **(F)** GSH-PX, **(G)** SOD, and **(H)** T-AOC levels in serum from diabetics rats were determined using relevant kits. (Means ± SEM; n = 6; *P < 0.05, **P < 0.01, ***P < 0.001, ****p<0.0001 vs. Model group; #P < 0.05 vs. positive group).

To further elucidate puffball spore effects on oxidative damage, ROS production and MDA, 8-oxo-dG, CAT, GSH-Px, SOD, and T-AOC secretion were detected. As shown in **Figure 4A** and partly quantified in **Figure 4B**, ROS production in the model group had the highest fluorescence intensity; however, it was reduced after treatment with puffball spores. MDA and 8-oxo-dG are indices of peroxidation and oxidative stress in diabetes. Puffball spores effectively reduced oxidative damage (**Figures 4C, D**). **Figures 4E–H** shows that the puffball spores increased the activities of CAT, SOD, GSH-Px and improved the antioxidant capacity, thus restoring the balance between oxidative and antioxidative processes.

### Puffball spores activate Akt/Nrf2 signal transduction and promote the expression of its downstream antioxidant genes to promote wound healing

**Figure 5:**
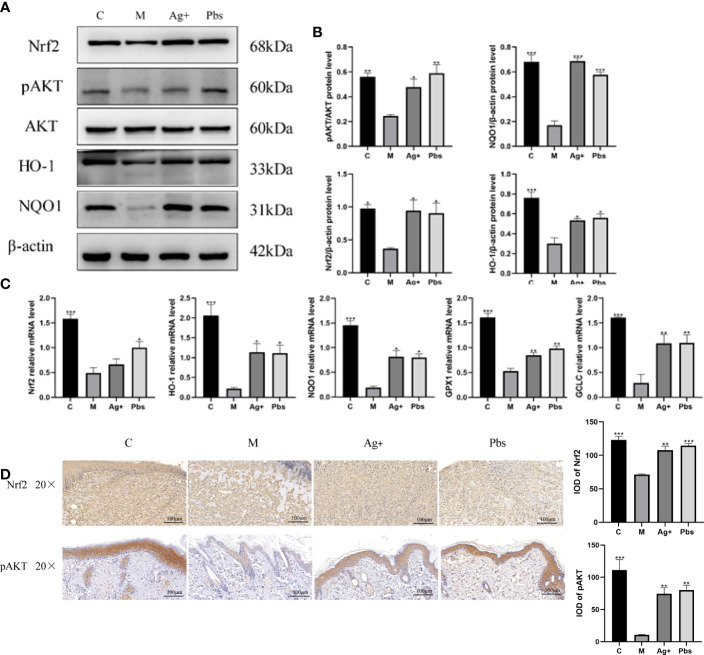
Puffball spores activates Akt/Nrf2/HO-1 signal transduction to promote wound healing **(A, B)** Protein levels of AKT, pAKT, Nrf2, NQO1, and HO-1 protein levels in wound tissue. **(C)** Nrf2, NQO1, GCLC, GPX1, and HO-1 gene expression in wound tissue. (Means ± SEM, n = 3; *P < 0.05, **P < 0.01, ***P < 0.001 vs. Model group). **(D)** Nrf2, pAKT expression in wound tissues was detected by immunohistochemistry (scale bar = 100 µm; magnification, 200×; n = 3).

Nrf2 plays an important role in regulating cellular redox homeostasis and functions. Nrf2 is activated in response to endogenous and exogenous stress. This activation induces the expression of antioxidant, cytoprotective, and detoxification genes expression (15, 16). Given that the Akt/Nrf2/HO-1 pathway exerts important protective effects against diabetes through the enhancement of the tissue’s anti-oxidative stress ability, we investigated whether puffball spores are engaged in the Akt/Nrf2/HO-1 pathway regulation in wound healing. The western blot (**Figures 5A, B**) results showed that puffball spores upregulated pAKT, Nrf2, NQO1, and HO-1 expression. The RT-qPCR results (**Figure 5C**) showed that the puffball spores upregulated Nrf2, NQO1, GCLC, GPX1, and HO-1 gene expression. The immunohistochemistry results (**Figure 5D**) showed that the expression of pAkt and Nrf2 in the wound tissue was significantly increased after treatment with puffball spores, which was consistent with the results of western blot.

## Discussion

Nrf2 is a transcription factor that regulates the expression of hundreds of genes involved in cell defense against oxidative stress (17). Nrf2 binds to antioxidative response elements (AREs), inducing the transcription of related antioxidant enzymes, such as SOD, HO-1, NQO-1 and CAT (18). In numerous studies, Nrf2 activation did not reduce oxidative stress or inflammation in normal settings, in contrast to the demonstrated benefits of Nrf2 activation in diabetes patients (19, 20). This provides an effective approach to treat chronic diabetic wounds. In long-term hyperglycemia, there is a delay in diabetic wound healing, accompanied by severe oxidative stress, increased inflammatory infiltration, and weak angiogenesis. Our results revealed that treatment with puffball spores enhanced pAKT, Nrf2, HO-1, and NQO1 expression in the wound tissue. Furthermore, we also observed that treatment with puffball spores elevated the SOD, CAT, GSH-Px, and T-AOC levels in diabetic rat. These results suggested that puffball spores could improve the antioxidant capacity of diabetic wounds, achieving a balance between oxidation and antioxidantion process during wound healing. Meanwhile, our study showed that the puffball spores could reduce the levels of proinflammatory related factors IL-1β and TNF-α, and increase the expression of VEGF in serum and CD31 in wound tissues. These results indicate that puffball spores work *via* a pharmacological mechanism whereby they reduce inflammatory reactions and promote angiogenesis.

Current reports on the treatment of diabetes wounds by puffball spores do not mention a its specific mechanism. Thus, the present study preliminarily discussed the potential pharmacological mechanism of puffball spores. *In vivo* experiments demonstrated that puffball spores accelerated diabetic wound healing by activating the AKT/Nrf2/HO-1 signaling pathway, attenuating oxidative stress, reducing inflammatory infiltration, and enhancing angiogenesis. Furthermore, in a diabetic rat model of chronic wound healing, puffball spores notably increased angiogenesis and suppressed both inflammatory infiltration and collagen deposition by reducing the levels of MDA and 8-oxo-dG and enhancing the activities of SOD, CAT, and GSH-Px and the T-AOC.

To the best of our knowledge, our study is the first to demonstrate a novel function of puffball spores during the healing of DFUs. Puffball spores accelerated wound healing by activating Akt/Nrf2 and promoting the expression of its downstream antioxidant genes, achieving considerable antioxidant activity. Moreover, puffball spores modulated the inflammatory response by regulating the release of inflammation related factors (IL-6, IL-1β, and TNF-α). Furthermore, they improve the deposition of collagen fibers and enhanced angiogenesis, providing suitable conditions to promote wound healing. Thus, the present study lays the theoretical groundwork for further clinical research and application of puffball spores in the treatment of DFUs.

## Data availability statement

The datasets presented in this study can be found in online repositories. The names of the repository/repositories and accession number(s) can be found in the article/supplementary material.

## Ethics statement

The animal study was reviewed and approved by The Animal Ethics Committee of the Nanjing University of Chinese Medicine approved the animal experiments (No. 202009A034).

## Author contributions

WM, GH and TH were mainly responsible for designing the experimental content. PS, BL, and SW completed animal experiments. PF tested biochemical indicators. JC finished searching relevant documents. WM and GH controlled the quality audit and acted as the corresponding authors. All authors contributed to the article and approved the submitted version.

## Funding

This study was supported by Huang Guicheng: National Famous Old Chinese Medicine Experts Inheritance studio Construction Project, the National Administration of Traditional Chinese Medicine: Young Qihuang Scholar Talent Project, Research and Practice Innovation Plan for Postgraduates of Jiangsu (KYCX22_1918), National key research and development plan project: Research on modernization of traditional Chinese Medicine (2019YFC1709905).

## Acknowledgments

The authors thank Manlu Fan for her help in the immunohistochemical experiment.

## Conflict of interest

The authors declare that the research was conducted in the absence of any commercial or financial relationships that could be construed as a potential conflict of interest.

## Publisher’s note

All claims expressed in this article are solely those of the authors and do not necessarily represent those of their affiliated organizations, or those of the publisher, the editors and the reviewers. Any product that may be evaluated in this article, or claim that may be made by its manufacturer, is not guaranteed or endorsed by the publisher.

## References

[1] Lima AL Illing T Schliemann S Elsner P. Cutaneous manifestations of diabetes mellitus: A review. Am J Clin Dermatol (2017) 18(4):541–53. doi: 10.1007/s40257-017-0275-z 28374407

[2] Yang Y Zhang B Yang Y Peng B Ye R. FOXM1 accelerates wound healing in diabetic foot ulcer by inducing M2 macrophage polarization through a mechanism involving SEMA3C/NRP2/Hedgehog signaling. Diabetes Res Clin Pract (2021) 184:109121. doi: 10.1016/j.diabres.2021.109121 34742786

[3] Armstrong DG Boulton AJM Bus SA. Diabetic foot ulcers and their recurrence. N Engl J Med (2017) 376(24):2367–75. doi: 10.1056/NEJMra1615439 28614678

[4] Zhang F Liu Y Wang S Yan X Lin Y Chen D. Interleukin-25-Mediated-IL-17RB upregulation promotes cutaneous wound healing in diabetic mice by improving endothelial cell functions. Front Immunol (2022) 13:809755 doi: 10.3389/fimmu 35126394 PMC8810642

[5] Zhao X Xu M Tang Y Xie D Wang Y Chen M. Changes in miroRNA-103 expression in wound margin tissue are related to wound healing of diabetes foot ulcers. Int Wound J (2022). doi: 10.1111/iwj PMC9885465 35837786

[6] Wan X Chen Y Geng F Sheng Y Wang F Guo J. Narrative review of the mechanism of natural products and scar formation in wound repair. Ann Transl Med (2022) 10(4):236. doi: 10.21037/atm-21-7046 35280378 PMC8908149

[7] Ali R Khamis T Enan G El-Didamony G Sitohy B Abdel-Fattah G. The healing capability of clove flower extract (CFE) in streptozotocin-induced (STZ-induced) diabetic rat wounds infected with multidrug resistant bacteria. Molecules (2022) 27(7):2270. doi: 10.3390/molecules27072270 35408668 PMC9000752

[8] Li Q Liang S Lai Q Shen L Zhang Y Guo R. Heme oxygenase-1 alleviates advanced glycation end product-induced oxidative stress, inflammatory response and biological behavioral disorders in rat dermal fibroblasts. Exp Ther Med (2021) 22(5):1212. doi: 10.3892/etm.2021.10646 34584557 PMC8422385

[9] Teng M Li Z Wu X Zhang Z Lu Z Wu K. Development of tannin-bridged cerium oxide microcubes-chitosan cryogel as a multifunctional wound dressing. Colloids Surf B Biointerf (2022) 214:112479. doi: 10.1016/j.colsurfb.2022.112479 35349942

[10] Ogbole OO Nkumah AO Linus AU Falade MO. Molecular identification, *in vivo* and *in vitro* activities of calvatia gigantea (macro-fungus) as an antidiabetic agent. Mycology (2019) 10(3):166–73. doi: 10.1080/21501203.2019.1595204 PMC6691841 31448150

[11] Zeng Q Singh R Ye Y Cheng S Fan C Zeng Q. Calvatia lilacina extracts exert anti-Breast-Cancer bioactivity through the apoptosis induction dependent on mitochondrial reactive oxygen species and caspase activation. Nutr Cancer (2022) 74(3):1058–70. doi: 10.1080/01635581.2021.1936576 34121543

[12] Lee S Lee D Lee JC Kang KS Ryoo R Park HJ. Bioactivity-guided isolation of anti-inflammatory constituents of the rare mushroom calvatia nipponica in LPS-stimulated RAW264. 7 Macrophages Chem Biodivers (2018) 15(9):e1800203. doi: 10.1002/cbdv.201800203 29933520

[13] Petrovic' P Vunduk J Klaus A Kozarski M Bugarski B. Biological potential of puffballs: A comparative analysis. J Funct Foods (2016) 21:36–49. doi: 10.1016/j.jff.2015.11.039

[14] Long M Rojo de la Vega M Wen Q Bharara M Jiang T Zhang R. An essential role of NRF2 in diabetic wound healing. Diabetes (2016) 65(3):780–93. doi: 10.2337/db15-0564 PMC4764153 26718502

[15] Kahroba H Shirmohamadi M Hejazi MS Samadi N. The role of Nrf2 signaling in cancer stem cells: From stemness and self-renewal to tumorigenesis and chemoresistance. Life Sci (2019) 239:116986. doi: 10.1016/j.lfs.2019.116986 31678283

[16] Jeddi F Soozangar N Sadeghi MR Somi MH Samadi N. Contradictory roles of Nrf2/Keap1 signaling pathway in cancer prevention/promotion and chemoresistance. DNA Repair (Amst) (2017) 54 13–21. doi: 10.1016/j.dnarep.2017.03.008 28415030

[17] Ulasov AV Rosenkranz AA Georgiev GP Sobolev AS. Nrf2/Keap1/ARE signaling: Towards specific regulation. Life Sci (2022) 291:120111. doi: 10.1016/j.lfs.2021.120111 34732330 PMC8557391

[18] Basu P Averitt DL Maier C Basu A. The effects of nuclear factor erythroid 2 (NFE2)-related factor 2 (Nrf2) activation in preclinical models of peripheral neuropathic pain. Antioxid (Basel) (2022) 11(2):430. doi: 10.3390/antiox11020430 PMC8869199 35204312

[19] Sun W Liu X Zhang H Song Y Li T Liu X. Epigallocatechin gallate upregulates NRF2 to prevent diabetic nephropathy *via* disabling KEAP1. Free Radic Biol Med (2017) 108:840–57. doi: 10.1016/j.freeradbiomed.2017.04.365 28457936

[20] Dong W Jia Y Liu X Zhang H Li T Huang W. Sodium butyrate activates NRF2 to ameliorate diabetic nephropathy possibly *via* inhibition of HDAC. J Endocrinol (2017) 232(1):71–83. doi: 10.1530/JOE-16-0322 27799462

